# Identification of Anthocyanin Composition and Functional Analysis of an Anthocyanin Activator in *Solanum nigrum* Fruits

**DOI:** 10.3390/molecules22060876

**Published:** 2017-05-25

**Authors:** Shaoli Wang, Zhaohui Chu, Mingxing Ren, Ru Jia, Changbao Zhao, Dan Fei, Hao Su, Xiaoqi Fan, Xiaotian Zhang, Yang Li, Yingzi Wang, Xinhua Ding

**Affiliations:** 1 State Key Laboratory of Crop Biology, Shandong Provincial Key Laboratory for Biology of Vegetable Diseases and Insect Pests, Shandong Agricultural University, Taian 271018, Shandong, China; Shaoliwang123@126.com (S.W.); zchu@sdau.edu.cn (Z.C.); rjia1104@163.com (R.J.); bnj1314@163.com (C.Z.); sdauzbsh@163.com (H.S.); 17863800822@163.com (X.F.); sdxtian@126.com (X.Z.); younuowanwan@126.com (Y.L.); 2 Shaoxing Entry-Exit Inspection and Quarantine Bureau, Shaoxing 312000, Zhejiang, China; mxren_sx@126.com; 3 Anhui Biothun Biotechnology Company, Hefei 230088, Anhui, China; dannyfei@126.com; 4 Institute of Plant Protection, Yantai Academy of Agricultural Science, Yantai 265500, Shandong, China; ytnkyzbs@126.com

**Keywords:** *Solanum nigrum*, anthocyanin, HPLC-MS/MS, antioxidant capacity, SnMYB transcription factor

## Abstract

*Solanum nigrum* fruits have been conventionally used in beverages due to their nutritional substances such as minerals, vitamins, amino acids, proteins, sugars, polyphenols, and anthocyanins. The characterization of components and regulatory mechanism of anthocyanins in *S. nigrum* fruits have rarely been reported. In this study, we determined that the peel and flesh of *S. nigrum* fruits shared similar HPLC profiles but different contents and total antioxidant activities for anthocyanins. After an efficient purification method, mainly including extraction with pH 1.0 distilled water and then desorption with pH 1.0 95% ethanol after a DM-130 resin adsorption step to obtain more pure anthocyanin extracts, the purity of anthocyanins extracted from *S. nigrum* fruits reached 56.1%. Moreover, eight anthocyanins from *S. nigrum* fruit were identified with HPLC-MS/MS for the first time. A typical R2R3-MYB transcription factor gene, *SnMYB*, was also cloned for the first time by rapid amplification of cDNA ends (RACE)-PCR from *S. nigrum*. Moreover, the contents of anthocyanins were shown to correlate well (*r* = 0.93) with the expression levels of *SnMYB* gene during the fruit’s developmental stages. Most significantly, *SnMYB* gene successfully produced high anthocyanin content (1.03 mg/g) when *SnMYB* gene was transiently expressed in tobacco leaves. Taken together, *S. nigrum* fruits are a promising resource for anthocyanin extraction, and *SnMYB* gene is an activator that positively regulates anthocyanin biosynthesis in *S. nigrum*.

## 1. Introduction

As a group of natural pigments, anthocyanins are water-soluble and provide many flowers and fruits with their purple, blue, and red colors, which promotes pollination and seed distribution [1]. Natural anthocyanins have the ability to protect plants from biotic and abiotic stress [2]. For example, anthocyanins can provide plants with increased resistance to some fungal diseases and insect damage [3,4]. Furthermore, anthocyanins are capable of protecting plants from cold damage and UV irradiation [5]. In addition to the physiological effects in plants, a healthy diet rich in anthocyanins has a variety of notable health-promoting effects. Anthocyanins offer protection against certain chronic illnesses including hyperglycemia [6] and inhibit the growth of tumor cells in humans [7,8]. Anthocyanins also have been shown to improve vision [9]. Due to these benefits, anthocyanins are becoming increasingly commercial and have been utilized for use in beverages [10] and in therapy for many human diseases [6,7,8].

As an herbal plant, *S. nigrum* is known as “Black nightshade” or “Black stars”. In China, people have extensively used the plant for its therapeutic effects of inflammation due to its antipyretic and diuretic effects [11]. Furthermore, the *S. nigrum* fruits are rich in a variety of nutritional substances including minerals such as K, Na, Ca, Zn, and vitamins, amino acids, protein, sugars, polyphenols, and anthocyanins [12,13], so they are widely processed into edible products, such as juice, beverages, jam, fruit wine, and so on [10]. Mature fruits of *S. nigrum* are usually made into drinks to relieve tension and anxiety in Mexico [14]. The *S. nigrum* plant also plays a vital role in the remediation of soils contaminated with heavy metals, such as Cd and As [15].

Although there are many studies concerning anthocyanins in berries (cherry, blueberry, and blackcurrant) with red, blue, or purple colors, studies on the anthocyanins of *S. nigrum* fruits are rare. Furthermore, there are few studies regarding the purification and identification of anthocyanins in *S. nigrum* fruits. The purification of anthocyanins was investigated in different studies with different procedures. Strathearn et al. (2014) [16] increased the purity of anthocyanin extracts to 20.6% after an enrichment step in a C18 solid-phase extraction of blueberries. Liu et al. (2012) [17] utilized an Amberlite XAD-7 column and a Sephadex LH20 column to obtain a 31% purity of anthocyanin crude extracts of blueberries. Nonetheless, the purities of these abovementioned anthocyanin extracts were all below 40% in most studies.

Some endogenous MYB-bHLH-WD40 (MBW) complexes composed of R2R3-MYB, basic-Helix--Loop-Helix (bHLH), and WD40 transcription factors (TFs) are considered to participate in the regulation of anthocyanin biosynthesis [18]. Among them, R2R3-MYB TFs are thought to combine directly to the promoter regions of many structural genes related to anthocyanin biosynthesis [19]. However, intense anthocyanin accumulation occurred when exogenous R2R3-MYB was individually expressed in other plants. For example, kiwifruit AcMYB110 individually induced anthocyanin accumulation when ectopically expressed in tobacco leaves without ectopic expression of any other bHLH or WD40 as a partner [20]. Several R2R3-MYB anthocyanin activators have also been identified in *Solanaceae* plants. Ectopic expression of NtAn2 induced whole-plant anthocyanin production in tobacco and Arabidopsis [21]. Moreover, StAN1, StMYBA1, and StMYB113 were also found to be able to individually induce intense anthocyanin pigments when they were transiently expressed in tobacco leaves [22]. This evidence verifies that R2R3-MYB TFs are critically important for anthocyanin production. However, no MYB transcription factor associated with anthocyanin regulation has been cloned from *S. nigrum*.

Herein, we explored an efficient purification method to obtain purer anthocyanin extracts and identified the anthocyanin compositions of *S. nigrum* fruits with HPLC-MS/MS. In order to investigate the molecular mechanism of anthocyanin biosynthesis, we cloned an R2R3-MYB by rapid amplification of cDNA ends (RACE)-PCR and revealed the function of *SnMYB* gene by determining the relationship between *SnMYB* gene transcript levels and anthocyanin contents at different developmental stages of fruits, and we transiently expressed *SnMYB* gene to produce anthocyanins in tobacco leaves.

## 2. Results

### 2.1. Analyses of Anthocyanins and their Antioxidant Activity in S. nigrum Fruits

**Figure 1:**
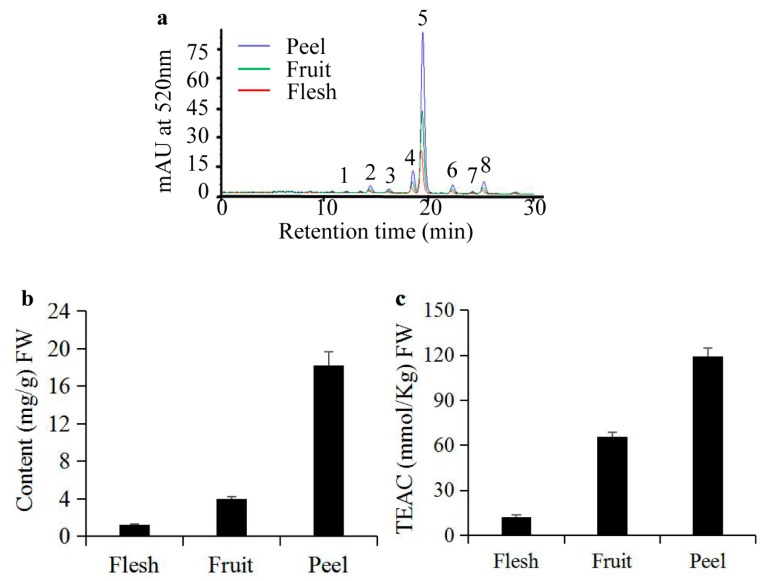
Different characteristics of anthocyanins from *S. nigrum* fruits. (**a**) HPLC chromatograms of anthocyanins in the peel, flesh, and the whole fresh fruit of *S. nigrum*; (**b**) Anthocyanin content; and (**c**) total antioxidant activities in the peel, flesh, and the whole fresh fruit of *S. nigrum*.

It has been reported that the peels of apples have greater antioxidant activity than does the flesh due to the presence of more anthocyanins and quercetin glycosides [23]. Therefore, we determined anthocyanin content and antioxidant activities of the peel, flesh, and whole fruit of S. nigrum. It was obvious that there were the same anthocyanin HPLC profiles among the peel, flesh, and whole fruit. The retention time of anthocyanin peaks were spread from 10 min to 25 min, approximately. Among them, peak five had the highest peak area (Figure 1a). The peel of S. nigrum fruit contains the highest anthocyanin content (18.215 mg/g Fresh Weight (FW)). In contrast, the anthocyanin content was the lowest (1.263 mg/g FW) in the flesh. Anthocyanin content in the whole fruit (3.954 mg/g FW) of S. nigrum was between that of the peel and the flesh (Figure 1b). Corresponding to the anthocyanin contents, there was the same trend of antioxidant activities in the peel, flesh, and whole fruit with the Trolox equivalent antioxidant capacity (TEAC) measurements of 113.47 ± 2.48, 12.12 ± 0.79, and 63.74 ± 0.34 mmol/kg FW, respectively (Figure 1c). It it worth noting that the anthocyanin content in the peel, flesh, and whole fruit are also strongly linearly related to the TEAC measurements, as shown by the Pearson coefficient, S. nigrum r = 0.93, which is consistent with reports in many plants with high anthocyanin content [24,25,26].

### 2.2. Purification of Anthocyanins from S. nigrum Fruits

**Figure 2:**
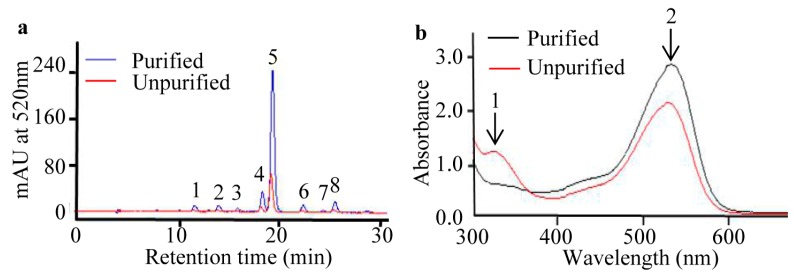
Different characteristics of purified and unpurified anthocyanin extracts from *S. nigrum* fruits. (**a**) HPLC chromatograms of purified and unpurified anthocyanins from *S. nigrum* fruits; (**b**) Spectral characters of purified and unpurified anthocyanins from *S. nigrum* fruits. 1: absorption peak of hydroxycinnamate; 2: absorption peak of anthocyanins.

In light of abovementioned analyses of anthocyanin content and antioxidant activities of *S. nigrum* fruits, we applied a purification method to obtain purer anthocyanin extracts. HPLC profiles indicate that there are no obvious changes in anthocyanin HPLC profiles, but there is a higher HPLC signal for the purified than for that of the unpurified anthocyanin extract from *S. nigrum* fruits (Figure 2a). Next, the purities of both purified and unpurified anthocyanin extracts were determined by spectroscopic scanning. The spectral character of the unpurified sample demonstrates the highest peak at 520 nm (peak 2) which is confirmed as the anthocyanin absorption peak, and a lower peak at 320 nm (peak 1) which is recognized as the hydroxycinnamate absorption peak [27]. After the purification steps, however, the absorption peak at 520 nm of purified anthocyanin extract is higher than that of unpurified anthocyanin extract, and the peak at 325 nm is lower in the opposite manner (Figure 2b), which means our method is conducive to eliminating non-anthocyanin components. The purity of purified anthocyanin extract powder (5.99 g) from *S. nigrum* fresh fruits (1 kg) is increased to 56.1% from 0.395% of the whole fresh fruit. Accordingly, the antioxidant capacity of the purified anthocyanin extract is increased to 773.54 mmol/kg Dry Weight (DW) from 63.74 mmol/kg FW of the whole fresh fruit. These results suggest our purification method is promising for obtaining high-purity anthocyanins from *S. nigrum* fruits.

### 2.3. Identification of Anthocyanin Composition by Mass Spectrometry

**Figure 3:**
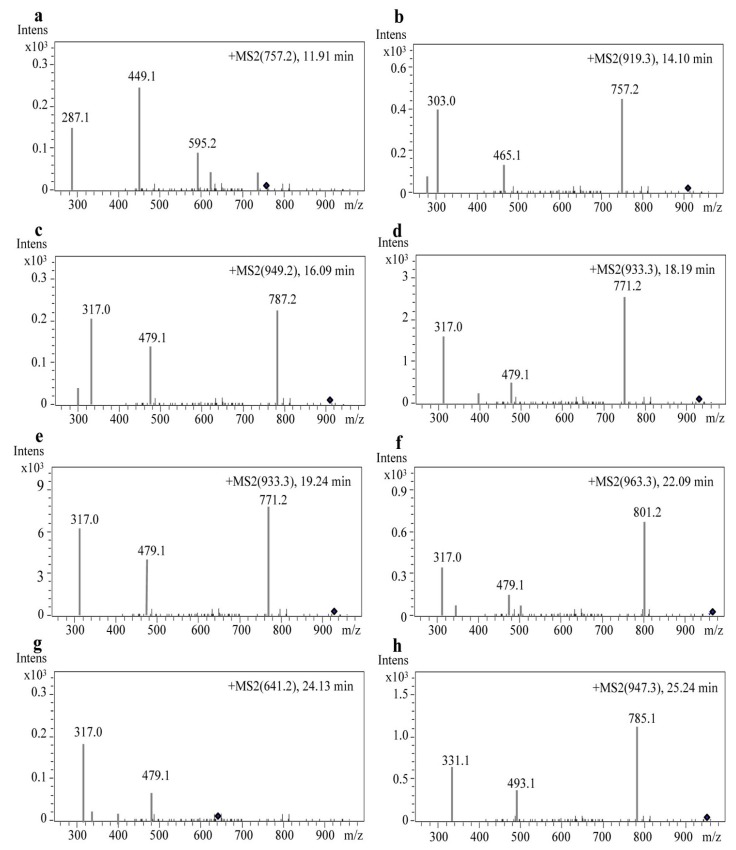
Mass spectrometric data of eight anthocyanins in *S. nigrum* fruits. (**a**) Peak 1: Cyanidin-3-rutinoside-5-glucoside; (**b**) Peak 2: Delphinidin-3-(*p*-coumaroyl)-rutinoside-5-glucoside; (**c**) Peak 3: Petunidin-3-*O*-rutinoside-(caffeoyl)-5-*O*-glucoside; (**d**) Peak 4: Petunidin-3-(*cis*-*p*-coumaroyl)-rutinoside-5-glucoside; (**e**) Peak 5: Petunidin-3-(*trans*-*p*-coumaroyl)-rutinoside-5-glucoside; (**f**) Peak 6: Petunidin-3-(feruloyl)-rutinoside-5-glucoside; (**g**) Peak 7: Petunidin-3-*O*-glucoside-5-*O*-glucoside; (**h**) Peak 8: Malvidin-3-(*p*-coumaroyl)-rutinoside-5-glucoside. Note: the black box indicates molecular weight of parent ion.

After HPLC separation, eight peaks (Figure 2a) of anthocyanin compounds were subsequently characterized by monitoring the molecular ion characteristics and referring to other literature [28,29,30,31]. The *m*/*z* ratio and HPLC-MS/MS ion graphs of each parent ion and their daughter fragments are shown in Table 1 and Figure 3. Four aglycones were determined as cyanidin aglycone (Cyd, *m*/*z* 287) [28], delphinidin aglycone (Dpd, *m*/*z* 303), petunidin aglycone (Ptd, *m*/*z* 317), and malvidin aglycone (Mv, *m*/*z* 331) [29]. Petunidin was the richest detected aglycone with five derivatives (peaks 3, 4, 5, 6, and 7) (Figure 2a; Table 1). Among them, peaks 4 and 5 (*m*/*z* 933) were different isomers, with two of the same daughter fragments (*m*/*z* 771 and *m*/*z* 479) (Table 1). The transitions 933–771 and 933–479 indicated a loss of glucose (*m*/*z* 162) and *p*-coumaroyl (*m*/*z* 454) [30] in peaks four and five, respectively. In addition, because the *cis*-*p*-coumaroyl derivative had higher polarity, it was eluted earlier than its trans configuration [29]. Therefore, peak four (Figure 3d) was tentatively recognized as petunidin-3-(*cis*-*p*-coumaroyl)-rutinoside-5-glucoside, and peak five (Figure 3e) was tentatively identified as petunidin-3-(*trans*-*p*-coumaroyl)-rutinoside-5-glucoside. Due to the petunidin aglycone (Ptd, *m*/*z* 317) and other two fragments (*m*/*z* 787 and *m*/*z* 479) identified in previous research [29], we supposed that peak three was petunidin-3-*O*-rutinoside-(caffeoyl)-5-*O*-glucoside. Transitions 963–801 and 963–479 indicated glucose (*m*/*z* 162) and feruloyl (*m*/*z* 484) [30] existed at peak six (*m*/*z* 963) (Figure 3f). Therefore, with a fragment of petunidin aglycone (Ptd, *m*/*z* 317), peak six was considered petunidin-3-(feruloyl)-rutinoside-5-glucoside. Peak seven (*m*/*z* 641) (Figure 3g) was the fifth peak with petunidin aglycone (Ptd, *m*/*z* 317). Along with the transition 641–479 leading to the loss of glucose (*m*/*z* 162), peak seven was identified as petunidin-3-*O*-glucoside-5-*O*-glucoside [31]. For the first anthocyanin elution component, peak one (*m*/*z* 757) (Figure 3a) was a unique anthocyanin with a cyanidin aglycone (*m*/*z* 287) fragment. In addition, transition 757–595 indicated a loss of glucose (*m*/*z* 162). This evidence suggests that peak one was cyanidin-3-rutinoside-5-glucoside [28]. By that analogy, transitions 919–757 and 919–465 implied a loss of glucose (*m*/*z* 162) and *p*-coumaroyl (*m*/*z* 454) at peak two (*m*/*z* 919) (Figure 3b), and the delphinidin aglycone (Dpd, *m*/*z* 303) revealed that peak two was delphinidin-3-(*p*-coumaroyl)-rutinoside-5-glucoside. As the last eluted anthocyanin, peak eight (*m*/*z* 947) (Figure 3g) should have a loss of glucose (*m*/*z* 162) and *p*-coumaroyl (*m*/*z* 454) due to its two daughter ions (*m*/*z* 785 and *m*/*z* 493) (Figure 3g). Like peaks one and two, peak eight was also unique, having the fragment of malvidin aglycone (Mv, *m*/*z* 331), which indicated that peak eight was malvidin-3-(*p*-coumaroyl)-rutinoside-5-glucoside.

### 2.4. Cloning of SnMYB from S. nigrum

**Figure 4:**
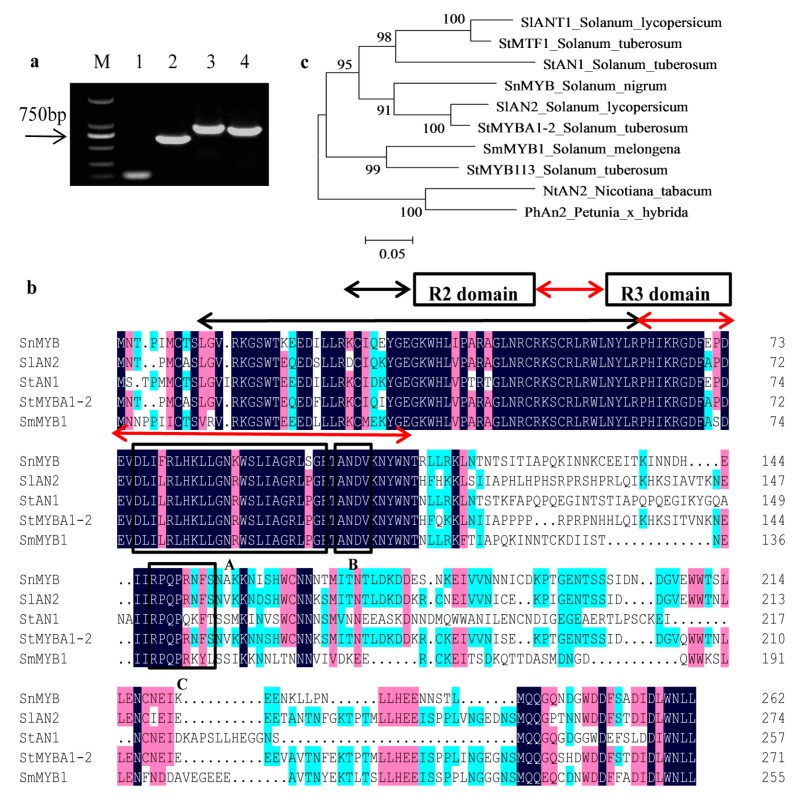
Isolation of *SnMYB* and sequence alignment with other anthocyanin R2R3-MYB regulators from *Solanaceae* plants. (**a**) Cloning of *SnMYB* gene with rapid amplification of cDNA ends (RACE)-PCR. M: DL2000 DNA marker; lane 1: a 115-bp fragment of conserved region; lane 2: a 564-bp 5′ cDNA fragment with 5′RACE; lane 3: an 844-bp 3′ cDNA fragment with 3′RACE; lane 4: a 792-bp full-length coding sequence of *SnMYB*; (**b**) Protein sequence alignment of SnMYB with other anthocyanin-related MYB regulators from *Solanaceae* plants. The R2 and R3 repeat domains are indicated by black and red arrows, respectively. Box-A indicates the conserved region of the basic-Helix-Loop-Helix (bHLH) interacting motif ([DE]Lx2[RK]x3Lx6Lx3R). Box-B indicates a conserved motif [A/S/G]NDV in the R2R3 domain for dicot anthocyanin-promoting MYBs. Box-C indicates a C-terminal-conserved motif [R/K] Px[P/A/R]xx[F/Y] for anthocyanin-regulating MYBs; (**c**) Phylogenetic relationship analysis of SnMYB and known anthocyanin-related MYB regulators from other *Solanaceae* species. Sequences were aligned using DNAMAN version 4.0. Phylogenetic and molecular evolutionary analysis was carried out using MEGA version 5.1. The evolutionary history was inferred using the neighbor-joining method and 1000 bootstrap replicates.

To clarify the regulatory mechanism of anthocyanin biosynthesis in *S. nigrum* fruts, we intended to clone an R2R3-MYB transcription factor which functions in anthocyanin regulation. Therefore, the degenerate primers *SnMYB*-D-F/R were used for generating a 115-bp-conserved-region fragment (Figure 4a). Then, the conserved fragment was sequenced and used to design the gene-specific primers *SnMYB*-5′RACE-R and *SnMYB*-3′RACE-F to obtain a 564-bp 5′ cDNA fragment and an 844-bp 3′ cDNA fragment by rapid amplification of cDNA ends (RACE) following the manufacturer’s instructions of the SMART RACE cDNA amplification kit (Figure 4a). Finally, after the 5′-RACE and 3′-RACE fragments were assembled, *SnMYB*-FL-F and *SnMYB*-FL-R primers were designed to amplify a 792-bp coding sequence named *SnMYB* (Figure 4a). Subsequently, protein sequences of *SnMYB* and other R2R3-MYB TFs related to accumulation of anthocyanins in some Solanaceae plants were aligned with each other. It was discovered that there were highly conserved R2 and R3 MYB domains (Figure 4b) in the N-terminal region. Moreover, they all had other conserved motifs in the C-terminal region, such as the [D/E]Lx2[R/K]x3Lx6Lx3R motif (Box-A in Figure 4b), which is important for interaction with bHLH proteins [32], and the conserved ANDV motif (Box-B in Figure 4b) identified from MYB activators of the anthocyanins pathway in Rosaceae species [33]. In addition, the motif [R/K]Px[P/A/R]xx[F/Y] (Box-C in Figure 4b) is highly conserved in the anthocyanin-promoting MYBs of some plant species [34]. Furthermore, the phylogenetic analysis revealed that SnMYB was closely clustered with other anthocyanin-related MYBs from other Solanaceae species (Figure 4c).

### 2.5. Expression Analysis of SnMYB in Different Developmental Stages of S. nigrum Fruits

**Figure 5:**
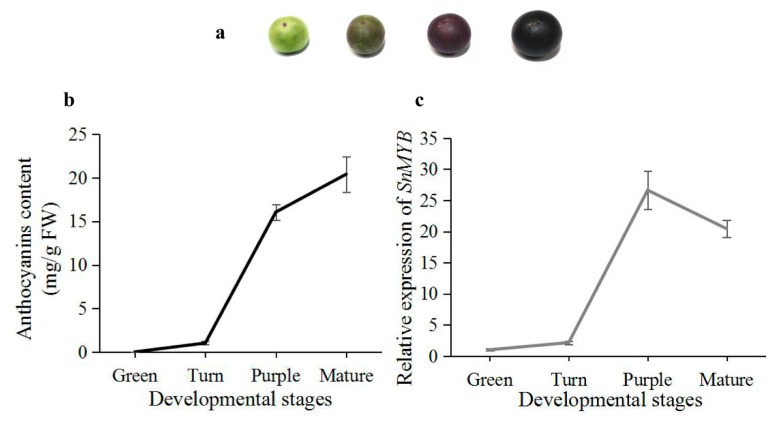
Correlation analysis of *SnMYB* expression with anthocyanin content in different developmental stages of the *S. nigrum* peel. (**a**) Color change with *S. nigrum* fruit maturation; (**b**) Determination of anthocyanin content; and (**c**) *SnMYB* expression levels in four developmental stages of the *S. nigrum* peel.

To identify the relationship between color deposition and transcript levels of SnMYB, we determined the anthocyanin contents and expression levels of SnMYB in different developmental stages. In addition, due to the highest anthocyanin content being in the peel, the experiment was carried out using the peel. Four different developmental stages, including the green, turn, purple, and mature stages, are found during fruit maturation. The green stage is a green color since it has few anthocyanins. However, other stages gradually become a deeper purple color with fruit maturation (Figure 5a). The mature stage has the highest anthocyanin content of all stages. The expression pattern of SnMYB displays a similar trend as the anthocyanin content in the four stages, except that the mature stage shows a slightly decreasing trend from the purple stage (Figure 5b).

### 2.6. Transient Expression of SnMYB in Tobacco Leaves

**Figure 6:**
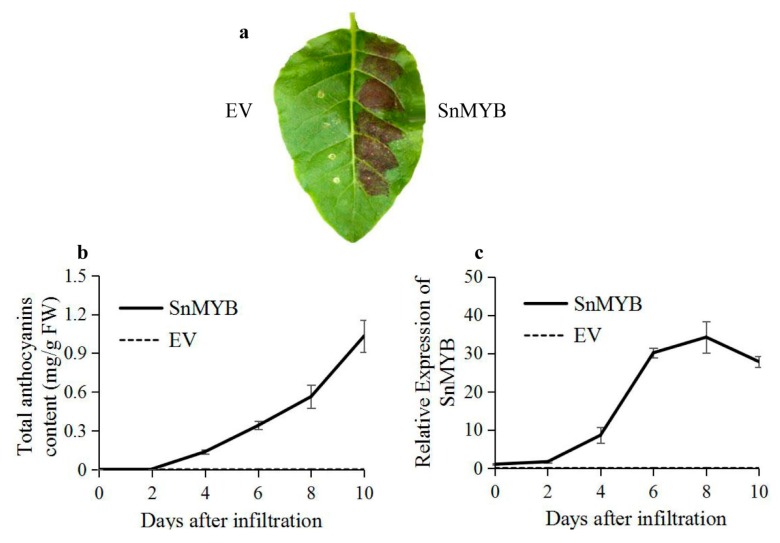
Transient activation of *SnMYB* in tobacco leaves. (**a**) Patch of anthocyanin production at the 8th day after infiltration; (**b**) Anthocyanin content; and (**c**) *SnMYB* expression levels in tobacco leaves infiltrated by *Agrobacterium*. The black solid line indicates infiltration with *SnMYB*, and the black dotted line indicates infiltration with the pGR106 empty vector (EV).

To further investigate the function of SnMYB as an anthocyanin regulator, we transiently expressed SnMYB in tobacco leaves. Four days after infiltration, red pigmentation was evident, and it developed into a dark red patch after 10 days (Figure 6a). Accordingly, the anthocyanins reached up to the maximum contents (1.03 mg/g) at 10 days after infiltration, which was even higher than that of blueberry in other reports (0.41–0.83 mg/g) [35]. Moreover, the anthocyanin contents and relative expression levels of SnMYB were both measured after infiltration. Both displayed a sharply rising trend after 4 days, except there was a slightly descending trend of SnMYB expression beginning 8 days after infiltration (Figure 6b,c).

## 3. Discussion

In our study, the peel of *S. nigrum* fruits displayed the same HPLC profiles and higher anthocyanin content and antioxidant activity than that of the flesh and whole fresh fruit (Figure 1), which is consistent with other reports [23,36]. Moreover, it is worth noting that the HPLC profile and content of anthocyanins in *S. nigrum* fruits were different and higher than those of other report [12]. These differences were most likely caused by different extraction methods [37] and raw materials used [35]. We found that there existed a strong positive correlation, with correlation coefficient: *r* = 0.93, between anthocyanin content and antioxidant capacities of the peel, flesh, and whole fresh fruit. These results indicate that anthocyanins are the major contributor to the total antioxidant capacity of *S. nigrum* fruit, which is consistent with previous reports [25,26]. Until now, most anthocyanin extractions and purifications have been carried out with blueberries or blackcurrants but not with *S. nigrum* fruits, and most purities were all below 40% [16,17], which were also lower than our result of purified anthocyanins from *S. nigrum* fruits. This evidence indicates that our purification method provided a fair chance of extracting anthocyanins from *S. nigrum* fruits with no change in composition but a large increase in purity.

In our study, we succeeded in identifying four anthocyanin aglycones by HPLC-MS/MS, including cyanidin (Cy, peak 1), malvidin (Mv, peak 8), petunidin (Pt, peaks 3, 4, 5, 6, and 7) and delphinidin (Dp, peak 2), in *S. nigrum* fruits (Table 1). Among the four anthocyanin aglycones, petunidin (Pt) aglycone was the most abundant component, with five derivatives (peaks 3, 4, 5, 6, and 7) (Table 1). The highest peak, five, was identified as petunidin-3-(*trans*-*p*-coumaroyl)-rutinoside-5-glucoside, which was in agreement with results in *Lycium ruthenicum* Murr [29] and tomato [30], also belonging to the *Solanaceae* genus. Interestingly, a cyanidin anthocyanin aglycone (peak 1) (Table 1) was firstly obtained in our result but not in other research on anthocyanins from *S. nigrum* fruits or other *Solanaceae* species [12]. It may be that the mass spectrometer used in this study has a higher sensitivity.

For the first time, we cloned an anthocyanin-related R2R3-MYB gene with RACE-PCR in the case of unknown genome sequences of *S. nigrum*. Three motifs including [D/E]Lx2[R/K]x3Lx6Lx3R [32], ANDV [33], and [R/K]Px[P/A/R]xx[F/Y] [34] displayed in the SnMYB protein sequence. With analysis of the phylogenetic relationship between SnMYB and other anthocyanin-activating MYBs of *Solanaceae* plants, we concluded that SnMYB probably has the capability of being an R2R3-MYB which could activate anthocyanin production in *S. nigrum* fruits.

In addition, there was a positive correlation, with *r* = 0.93, between the *SnMYB* transcript levels and anthocyanin accumulation (Figure 5), which was consistent with the result of *MYB* genes and anthocyanin accumulation in apples [38]. More importantly, the *SnMYB* gene can produce anthocyanins when transiently expressed in tobacco leaves at four days after infiltration (Figure 6a). The anthocyanin content reached the maximum content (1.03 mg/g) at 10 days after infiltration with *SnMYB*, which was even higher than that of blueberries (0.41–0.83 mg/g) [35] and was similar to the anthocyanin content of *AcMYB110* (0.6–1.4 mg/g) [39] and *StAN1-R1* (0.75–1.63 mg/g) [22] expressed in tobacco leaves. This result revealed that *SnMYB* most likely possesses a similarly strong function as *AcMYB110* and *StAN1-R1* in activating anthocyanin production when *SnMYB* was expressed in other plants to produce anthocyanins.

## 4. Materials and Methods

### 4.1. Plant Materials

Fruits of *S. nigrum* cultivar SN-0013 were harvested in a greenhouse. Whole fruits of different developmental stages were integrally harvested, frozen in liquid nitrogen, and stored at −80 °C until used for extraction of anthocyanins and RNA for further experiments.

### 4.2. HPLC and Mass Spectrometric Identification of Anthocyanins

Samples were ground into powders (approximately 0.2 g) in a mortar filled with liquid nitrogen. Next, the extraction method of Butelli et al. (2008) [40], with slight modifications, was used. The powder was extracted with 2 mL of 100% MeOH (Sigma, St. Louis, MI, USA). The powder/solvent mixture was stored at 4 °C for 12 h and shaken every 15 min in the first 2 h, avoiding light exposure. Samples were centrifuged at 2800 rpm for 30 min, and the supernatant was filtered through a 0.22 μm membrane filter. Then, samples were analyzed using an Agilent Technologies 1200 series HPLC (Agilent Technologies, Santa Clara, CA, USA) equipped with a diode array detector and a Zorbax Stablebond Analytical SB-C18 column (4.6 mm × 250 mm, 5 μm, Agilent Technologies, CA, USA). The mobile phase was as follows: solvent A consisted of 87% water, 11% acetonitrile (ACN), and 2% acetic acid, and solvent B consisted of 40% water, 58% ACN, and 2% acetic acid. The gradient elution was as follows: 0 min 4% B, 20 min 20% B, 35 min 40% B, 40 min 60% B, 45 min 90% B, and 55 min 4% B at a flow rate of 1 mL/min. The detection was recorded at 520 nm, and the column oven temperature was set to 30 °C. The anthocyanin standard (petunidin-3-(*trans*-coumaroyl)-rutinoside-5-glucoside) was obtained from the Anhui Biothun biotechnology company (Biothun, Anhui, China).

Purified anthocyanin extracts were dissolved in 100% MeOH and the mass spectrometric analysis was carried out on Q-TOF 5600 (Applied Biosystems, CA, USA) system in positive mode with *m*/*z* values ranging from 300 to 1000. Other Mass Spectrometry (MS) conditions were all as follows: nitrogen gas temperature, 550 °C; drying gas flow rate, 11 L/min; nebulization pressure, 35 psi; cone voltage, 40 V; capillary voltage, 3.5 kV.

### 4.3. Purification of Anthocyanins from S. nigrum Fruits

Whole frozen fruits (1 kg) were thawed and chopped into pieces. Then, they were extracted with 3 L of pH 1.0 distilled water adjusted with HCl overnight. The fruit juice supernatant was filtered with 200-mesh gauze to obtain the first crude juice. The precipitation of the pomace was then extracted using the above extraction solvent with ultrasonic wave treatment (500–700 W) three times every 30 min to obtain the second crude juice. Next, the two crude juices were loaded onto a DM-130 resin purchased from Amicogen (China) Biopham Co., Ltd. (Jining, China) (http://www.lksz.cn/). After the crude extracts were completely absorbed by the resin, pH 1.0 distilled water adjusted by HCl was applied to wash away impurities, such as free sugars and organic acids. Then, washing of the resin was stopped when the drip became clear. Afterwards, 95% ethanol (pH 3.0, adjusted by HCl) was used for eluting the purple anthocyanins from the resin until the drops were of achromatic color. At the end, the purple anthocyanin elution was dried into powder using a rotary evaporator (EYELA, OSB-2100-CE, Tokyo, Japan).

### 4.4. Purity Detection of Purified Anthocyanin Extracts

The method to determine the purity of anthocyanin extracts described by Chandra et al. (2001) [41] was used with modifications. Petunidin-3-(*trans*-coumaroyl)-rutinoside-5-glucoside as a standard and the purified anthocyanin powders were both dissolved in a pH 1.0 buffer solution containing 50 mM KCl and 150 mM HCl. Those solutions were added to a 10-mm path length cell to measure the absorbance at 520 nm and to generate a five-point calibration curve, with pH 1.0 buffer solution as a reference. However, beyond that, the absorption spectra of purified and unpurified anthocyanin extracts were recorded in the visible wavelength range from 300 to 600 nm [42]. All determinations were performed in triplicate using a Shimadzu UV-2401 PC spectrophotometer (Shimadzu, Kyoto, Japan).

### 4.5. Total Antioxidant Activity Assay

Samples were ground into powders (50 mg) in a mortar filled with liquid nitrogen. Then, the samples were both extracted with 70% MeOH. A stock solution was composed of 5 mL of 7 mmol/L ABTS (2.2′-azinobis [3-ethylbenzothiazoline-6-sulfonic acid]) and 88 µL of 140 mmol/L K_2_S_2_O_8_. Then, the stock solution was preincubated for at least 12 h in darkness to generate ABTS radical cations (ABTS^+^). ABTS^+^/Trolox (6-hydroxy-2,3,7,8-tetramethylchroman-2-carboxylic acid; Sigma) equivalent antioxidant capacity (TEAC) assays were performed to analyze the ability of samples to scavenge the ABTS radical cation (ABTS^+^) in relation to Trolox. We used different concentrations of Trolox to generate a calibration curve. All of the experiments were performed on a SpectraMax M2 (Molecular Devices, Sunnyvale, CA, USA) by measuring the absorbance at 735 nm. The results were expressed as millimole of Trolox equivalent antioxidant capacity (TEAC) per kilogram of fresh or dry weight [43].

### 4.6. RNA Extraction and Quantitative Real Time PCR

Different samples frozen at −80 °C were ground into powders with liquid nitrogen. Then, the total RNA was isolated from the powder using a Plant RNA Kit (OMEGA Bio-tek, Doraville, GA, USA). Subsequently, RNA samples were reverse-transcribed into complementary DNA using the Super-Quick RT MasterMix (CWBio, Beijing, China) following the manufacturer’s instructions. A CFX96 Real-Time System (Bio-Rad, Hercules, CA, USA) with SYBR Premix Ex Taq (TaKaRa, Dalian, China) were used for qRT-PCR. Reactions were performed in triplicate and contained 10 μL of master mix and each primer at 0.5 μM, 2 μL of diluted cDNA, and DNase-free water to a final volume of 20 μL. The PCR procedure was as follows: 1 cycle of 3 min at 95 °C, 40 cycles of denaturation for 15 s at 95 °C, annealing for 30 s at 60 °C, and elongation for 15 s at 72 °C. Afterwards, a melting curve analysis with continual fluorescence data acquisition during the 60–95 °C melting period was used to confirm there was only one product for each gene primer reaction [43]. The qRT-PCR primers of *SnMYB* and an internal standard *SnEF1α* are designed by Primer Premier 5, which are the primer pairs of *SnMYB qRT-F/R* (TCGAAACTTCTCAAACGCTAAGAA; TGTTGCTTTCGTCATCTTTGTCTAA) and *SnEF1α qRT-F/R* (TTTCACTGCCCAGGTCATCA; CAAACTTGACAGCAATGTGGGA). All experiments were carried out from three biological replicates and technical replicates.

### 4.7. SnMYB Gene Cloning and Protein Sequence Alignment

We queried different *MYB* genes involved in anthocyanin production from different *Solanaceae* plants to create degenerate primers named *SnMYB-D-F/R* (GAAGT(A/G/T)AG(A/G)AAAGG(A/G/T)CC(A/C)TGGA; GACCAGA(A/G/T)(A/G)TC(C/T)TCCAT(A/G)CTCCA), which were designed from the highly conserved regions of those *MYB* genes. Degenerate primers were used for amplifying partially conserved fragments of SnMYB from the first-strand cDNA synthesized using a HiFiScript cDNA Synthesis Kit (CWBio, Beijing, China). Then, the partially conserved fragment was sequenced, and gene-specific primers named *SnMYB*-5′RACE-R/*SnMYB*-3′RACE-F (TTTACCAGCTCTAGCAGGAATAAGATGC; CGAAAAAGTTGTAGACTGAGGTGGTTGA) were designed to amplify the cDNAs of SnMYB by rapid amplification of cDNA ends (RACE) in both the 5′ and 3′ directions following the manufacturer’s instructions of the SMARTer^®^ RACE 5′/3′ Kit (TaKaRa, Dalian, China). The fragments of the 5′-RACE and 3′-RACE of SnMYB were assembled and used to generate primers *SnMYB-FL-F*/*SnMYB-FL-R* (CCATCGATATGAATACTCCTATAATGTGTACGTCG; TTGCGGCCGCTTAATTAAGTAGATTCCATAGGTCAAT) to amplify the full-length coding sequence of *SnMYB*. The following PCR procedure was carried out with Lamp DNA polymerase (CWBio, Beijing, China) at 94 °C for 3 min, 35 cycles of 94 °C for 30 s, 55 °C for 30 s, 72 °C for 40 s, and a 10 min extension at 72 °C. All of the PCR products were cloned into a TA cloning vector named as pUC-T (CWBio, Beijing, China) for validation of DNA sequences. DNAMAN version 4.0 was used for aligning SnMYB amino acid sequences with some MYB anthocyanin activators from *Solanaceae* plants. The phylogenetic and molecular evolutionary analyses were carried out with MEGA version 5.1.

### 4.8. Transient Assays of SnMYB Function

We utilized the following processes to generate plasmids for transient expression assays in tobacco leaves. The amplification product of the primers *SnMYB-FL-F/SnMYB-FL-R* was digested with *Cla*I and *Not*I and then inserted into the plasmid pGR106 [44], which was digested with the same restriction enzymes and harbors the cauliflower mosaic virus (CaMV) 35S promoter. The recombinant vector was transformed into *Agrobacterium tumefaciens* GV3101 for infiltration into *Nicotiana tabacum* leaves which were grown in the greenhouse at 25 °C and 16/8 h light/dark. Details of the infiltration were the same as those described by Lim et al. (2012) [45]. In brief, *Agrobacterium tumefaciens* GV3101 with recombinant vector was grown at 28 °C and 220 rpm in luria-bertani (LB) medium including 50 µg/mL kanamycin, 10 mM 2-(4-Morpholino)ethanesulfonic acid (MES), and 20 µM Acetosyringone (AS). The bacteria were collected by 5000 rpm centrifugation at room temperature for 15 min. Then they were resuspended in MMA (10 mM MES, 10 mM MgCl_2_, 200 µM AS) solution to OD_600_ = 1.5 and then incubated at 28 °C for 3–5 h. The color change was monitored by digital images when the purple color appeared. In addition, three independent replicates of each infiltration were collected and frozen at −80 °C for subsequent analyses of anthocyanin content and relative *SnMYB* expression [39].

## 5. Conclusions

*S. nigrum* fruits are usually used as edible food for their nutritional substances such as minerals, vitamins, amino acids, proteins, sugars, polyphenols, and anthocyanins. In our study, we determined the anthocyanin content and identified the anthocyanin compositions by HPLC-MS/MS in *S. nigrum* fruits for the first time. High-purity anthocyanin extracts were obtained by establishing an efficient purification method, which is helpful for using *S. nigrum* fruits as a natural resource for anthocyanin extraction. It is most meaningful that we also isolated an anthocyanin-activating MYB transcription factor from *S. nigrum* for the first time, which is promising for breeding high anthocyanin-containing *S. nigrum* or other plant species with transgenic technology in the future.

## Figures and Tables

**Table 1:** Tentative identification of anthocyanin compositions in *S. nigrum* fruits.

Peak No.	Retention Time (min)	Anthocyanins	[M + H]^+^ (*m*/*z*)	Detected Fragments
1	11.921	Cyanidin-3-rutinoside-5-glucoside	757.2	595.22; 449.15; 287.08
2	14.109	Delphinidin-3-(*p*-coumaroyl)-rutinoside-5-glucoside	919.3521	757.27; 465.15; 303.08
3	16.093	Petunidin-3-*O*-rutinoside-(caffeoyl)-5-*O*-glucoside	949.2482	787.26; 479.15; 317.08
4	18.191	Petunidin-3-(*cis*-*p*-coumaroyl)-rutinoside-5-glucoside	933.3681	771.29; 479.16; 317.09
5	19.240	Petunidin-3-(*trans*-*p*-coumaroyl)-rutinoside-5-glucoside	933.3677	771.29; 479.17; 317.09
6	22.093	Petunidin-3-(feruloyl)-rutinoside-5-glucoside	963.3784	801.30; 479.16; 317.09
7	24.139	Petunidin-3-*O*-glucoside-5-*O*-glucoside	641.2	479.16; 317.09
8	25.242	Malvidin-3-(*p*-coumaroyl)-rutinoside-5-glucoside	947.3831	785.31; 493.18; 331.11
